# Transcriptomic population markers for human population discrimination

**DOI:** 10.1186/s12863-018-0663-2

**Published:** 2018-08-07

**Authors:** P. Daca-Roszak, M. Swierniak, R. Jaksik, T. Tyszkiewicz, M. Oczko-Wojciechowska, J. Zebracka-Gala, B. Jarzab, M. Witt, E. Zietkiewicz

**Affiliations:** 1 Institute of Human Genetics, Polish Academy of Sciences, Strzeszynska 32, 60-479 Poznan, Poland; 2 Maria Sklodowska-Curie, Memorial Cancer Center and Institute of Oncology, Gliwice Branch, Gliwice, Poland; 3 Present address: Laboratory of Human Cancer Genetics, Center of New Technologies, CENT, University of Warsaw, Warsaw, Poland; 4 Genomic Medicine, Medical University of Warsaw, Warsaw, Poland; 5 Institute of Automatic Control, Silesian University of Technology, Gliwice, Poland

**Keywords:** Gene expression study, Illumina platform, TLDA cards, Population-specific mRNA markers, Decision-tree, Classifier testing, Human population identification

## Abstract

**Background:**

Numerous studies have demonstrated significant differences in the expression level across continental human populations. Most of published results were performed on B-cell lines materials examined under specific laboratory conditions, without further validation in a primary biological material. The goal of our study was to identify mRNA markers characterized by a significant and stable difference in the gene expression profile in Caucasian and Chinese populations, both in the commercially available B-lymphocyte cell lines and in the primary samples of the peripheral blood.

**Results:**

The preliminary selection of population-differentiating transcripts was based on Illumina expression microarray analysis of the representative group of ethnically-specified B-lymphocyte cell lines. Twenty genes with the inter-population difference in the mean expression characterized by the at least 1.5-fold change and FDR <  0.05 were identified. Subsequently, a two-step validation procedure was carried out. In the first step, a subset of selected population- differentiating transcripts was tested in the independent set of B-lymphocyte cell lines, using TLDA cards. Based on TLDA analysis, three transcripts representing Fch > 2 were chosen for validation. The differentiating status was confirmed for all of them: *UTS2*, *UGT2B17* and S*LC7A7*. The mean expression of *UTS2* was higher in CHB (25.8-fold change compared to CEU), while the expression of *UGT2B17* and *SLC7A7* was higher in CEU (3.2- and 2.2-fold change, respectively).

In the next validation step, two transcripts were verified in the primary biological material. As an ultimate result of our study, two mRNA markers (*UTS2* and *UGT2B17*) exhibiting population differences in the expression level in both B-cell line and in the blood were identified. Further statistical analysis confirmed the discriminatory potential of these two markers.

**Conclusions:**

An inter-population differences on the level of gene expression were identified in both B-cell lines and peripheral blood samples. These findings may have a practical application in the field of forensic science. In particular, these transcripts, targeted by specific probes, may be used as population-specific targets in the efforts aiming to separate mixture of blood from individuals of different populations. Notwithstanding, these results have to be confirmed on extended population group.

**Electronic supplementary material:**

The online version of this article (10.1186/s12863-018-0663-2) contains supplementary material, which is available to authorized users.

## Background

The application of high throughput methods, like expression microarrays and next generation sequencing, targeting thousands of gene transcripts, has allowed exploration of the transcriptional variation in humans at the unprecedented scale. Numerous studies have demonstrated that, while the bulk of variation in the expression level is observed between individuals, significant differences across continental populations also exist [1–12].

In principle, the genes characterized by levels of expression that vary across different ethnic groups, may be used as markers for human population discrimination. In practice, it has to be remembered that for many genes, their expression profile is tissue specific and additionally depends on the environmental factors (e.g. diet, health, age etc.) [11, 13, 14]. In addition, many of the examples of population-specific expression profile have been detected in the studies based solely on B-lymphocyte-derived cell lines examined under specific laboratory conditions, without further validation in a primary biological material [1, 2].

**Fig. 1:**
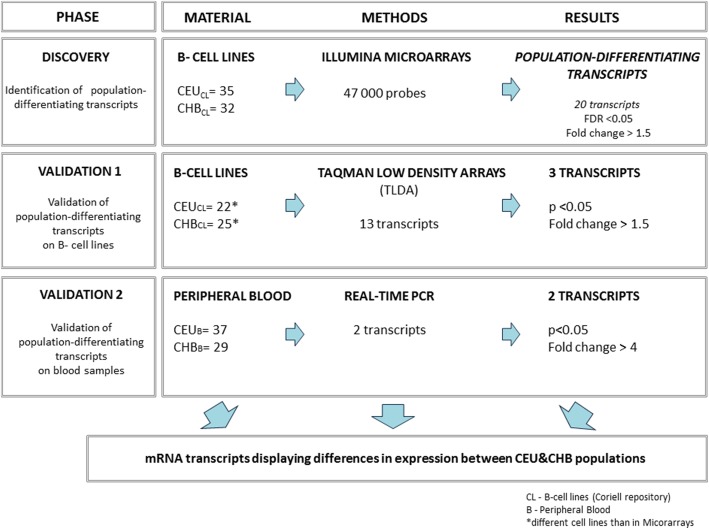
Study design

The goal of our study was to identify mRNA markers characterized by a significant and stable difference in the gene expression profile Caucasian and Chinese populations, both in the commercially available B-lymphocyte cell lines and in the primary samples of the peripheral blood (see Fig. 1).

To assess the population discriminating potential of two validated transcripts: *UTS2* and *UGT2B17*, three different binary classifiers were built and tested using 10-fold cross-validation method.

## Results

### Discovery phase: Identification of the differentially expressed genes

As a result of the supervised analysis of the Illumina expression microarray, 189 differentially expressed genes that met the FDR < 5% criterion were selected. Expression of twenty of those genes was characterized by the 1.5–2.5-fold population difference, including eleven genes with the increased and nine with the decreased expression in the CHB as compared to the CEU (see Table 1 for the details).

**Table 1:** A set of transcripts differentiating CEU and CHB cell lines in Illumina expression microarray

Genes with the higher expression in CHB				
Probe_ID	Symbol	*p*-value	FDR	Fold-change
5,270,541	*GAPDHL6* ^a^	2.09E-06	0.000526	2.48
6,290,228	*UTS2*	6.36E-16	2.37E-12	2.47
4,830,202	*CHI3L2*	9.78E-06	0.001106	2.37
7,400,193	*LOC729708* ^a^	4.72E-06	0.000734	1.77
6,420,168	*DBNDD2* ^a^	0.0020744	0.044493	1.66
770,564	*C1ORF115*	7.05E-06	0.000856	1.61
5,490,768	*GPR56* ^a^	2.12E-06	0.000526	1.60
6,650,242	*IFITM3*	3.50E-05	0.002848	1.58
1,990,672	*PLA2G4C*	2.80E-06	0.000581	1.56
3,370,730	*CDC42EP5*	0.000695	0.021436	1.55
2,060,181	*SNHG8* ^a^	0.0002602	0.011694	1.53
Genes with the lower expression in CHB				
Probe_ID	Symbol	*p* value	FDR	Fold-change
5,420,450	*UGT2B7*	1.02E-05	0.001123	0.41
3,850,168	*LOC644936* ^a^	0.0025063	0.049752	0.50
2,120,053	*CYP1B1*	0.0001846	0.009186	0.57
3,310,520	*MOXD1*	7.85E-05	0.005053	0.61
6,020,692	*HS.137971* ^a^	2.42E-09	2.26E-06	0.61
6,290,189	*UGT2B17*	1.29E-06	0.000402	0.64
4,830,632	*SLC7A7*	8.44E-05	0.005248	0.64
3,370,075	*S1PR4*	1.63E-07	7.60E-05	0.65
7,650,669	*TBC1D4*	1.14E-06	0.000387	0.65

^a^ *TLDA probe unavailable or unspecific*

### Validation 1: Expression of the population-differentiating transcripts in independent B-lymphocyte cell lines

The expression of 13 genes from 20 identified based on microarray study was further examined in the independent set of B-lymphocyte cell lines, using TLDA cards. Seven transcripts (see asterisks in Table 1), for which no specific TLDA probes were available, were not subjected to the validation.

Statistical analysis (comparison of the mean level of transcript abundance, represented by the relative quantification values) was performed in 12 of the 13 transcripts; *UGT2B7*, for which no amplification was obtained in any of the analyzed samples, was excluded from the statistical analysis.

Statistically significant (*p* <  0.05) population differences in the mean transcription level were observed in three out of the 12 analyzed genes: *UTS2*, *UGT2B17*, *SLC7A7* (Table 2).

**Table 2:** Validation of the population-differentiating transcripts on B-cell lines using TLDA cards

Gene name	Fold change	*p*-value
		U Mann Whitney/t-test*
Genes with higher expression in Chinese		
UTS2**	25.77	**< 0.00001**
CHI3L2	1.10	0.75656
C1ORF115	1.68	0.15560
IFITM3	1.62	0.58920
PLA2G4C	1.39	0.16152
CDC42EP5	1.13	0.75656
Genes with higher expression in European		
UGT2B7	did not amplify	
CYP1B1	1.64	0.23404
MOXD1	1.64	0.17702
UGT2B17**	3.23	**0.00350**
SLC7A7	2.17	**0.00120**
S1PR4	1.47	0.0960
TBC1D4	1.19	0.08012

*p-values for genes: *UTS2, UGT2B17, CHI3L2* and *C1orf115* which did not fulfill the requirement of normal distribution were tested using U-Mann Whitney statistics; other genes, were tested with using the t-test

**Validated on blood samples (see Validation 2 section). Significant population differences (*p* < 0.05) are indicated in bold

The greatest fold-change in the mean population level of expression was observed for *UTS2.* This transcript was ~ 25-times more abundant in CHB in comparison to CEU (*p* < 0.00001). On the contrary, transcripts of two other genes, *UGT2B17* and *SLC7A7,* were more abundant in CEU than in CHB population (the fold change values of ~ 3 and ~ 2; *p* = 0.00350 and *p* = 0.00120, respectively).

**Fig. 2:**
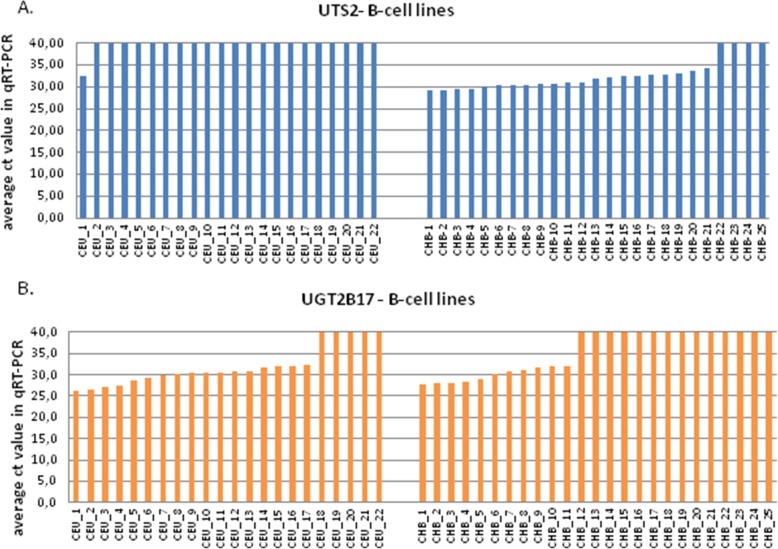
Average ct values obtained in qRT-PCR reactions for two tested genes: UTS2 (**a**) and UGT2B17 (**b**). Each bar represents B-cell line from Caucasian (left panel) and Chinese population (right panel)

Interestingly, the population differences in the mean level of expression of two best-differentiating genes, *UTS2* and *UGT2B17*, were caused by the complete lack of the corresponding transcripts amplification (ct ≥ 40 cycles) in individual samples rather than by the decreased level of their amplification in the whole set of population samples. The lack of the *UTS2* transcript amplification was noted in 21 of the 22 CEU, but only in 4 of the 25 CHB cell lines (see Fig. 2, panel a). The opposite was true for *UGT2B17*, where the lack of transcript amplification was seen in 14 CHB, but only in 5 CEU cell lines (see Fig. 2, panel b).

### Validation 2: Expression of the differentiating genes in the peripheral blood samples

**Fig. 3:**
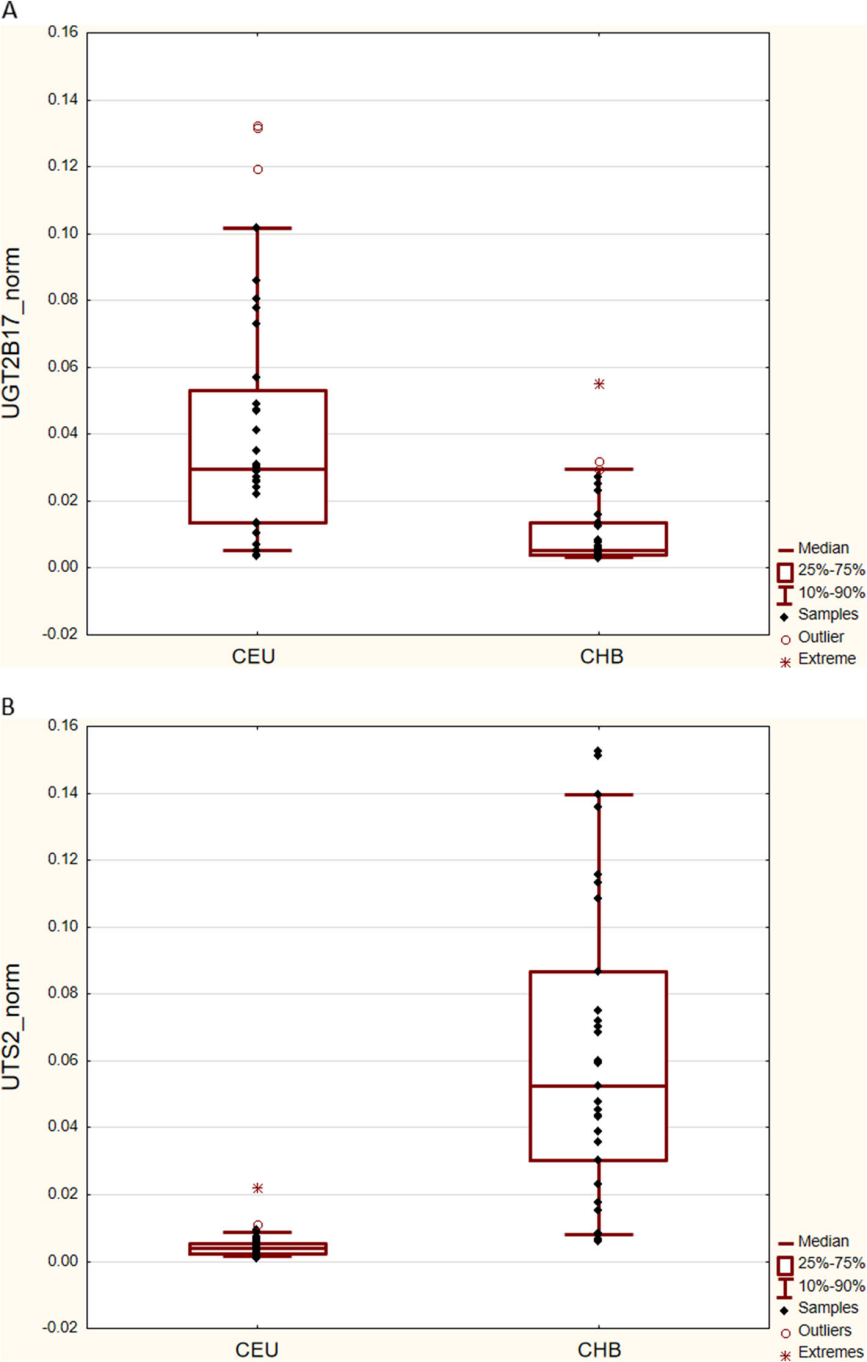
The normalized relative expression levels of *UGT2B17* (**a**) and *UTS2* (**b**) in the peripheral blood samples from Chinese (*n* = 29) and Caucasian (*n* = 37) males. Dots represent relative gene expression in the individual samples. The upper and lower edges of the boxes correspond to the first (Q1) and third (Q3) quartiles, respectively. The lines inside the boxes indicate the median expression values. The whiskers extend to the smallest and the largest observations within the 1.5-times interquartile range (IQR) from the box

To test, whether the differences in the abundance of transcripts observed between the CEU an CHB cell lines reflected real population differences in the gene expression (and were not due to the cell line peculiarities), the second step of validation was performed, using RNA isolated from the whole peripheral blood. Two best-differentiating transcripts, *UTS2* and *UGT2B17*, characterized by the at least 3-fold change in the mean expression level between CHB and CEU, were analyzed using 7900 HT Fast Real-Time PCR system. Thirty-seven of the RNA samples were from Caucasian and 29 from Chinese male donors. The expression of *UTS2* was 13 times higher (*p* < 0.001), while that of *UGT2B17* was 6 times lower (p < 0.001) in Chinese as compared to Caucasians, confirming the population differences observed in the 1st step of validation (Fig. 3).

**Fig. 4:**
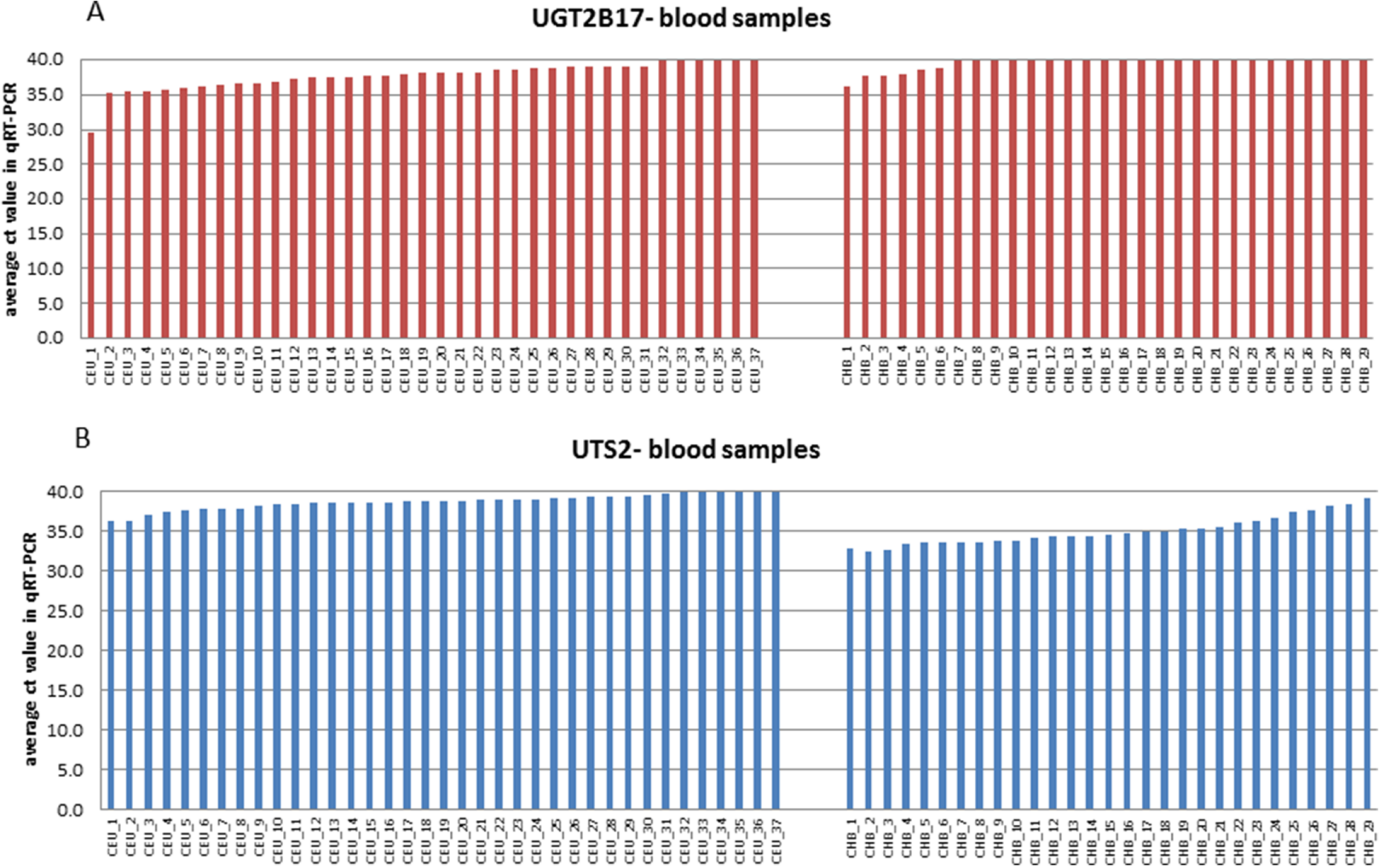
Average ct values obtained in qRT-PCR reactions for two tested genes: UGT2B17 (**a**) and UTS2 (**b**). Each bar represents blood sample from Caucasian (left panel) and Chinese population (right panel)

Similarly to the results obtained from the B-cell lines, the inter-population differences in the mean level of *UGT2B17* expression in blood were caused by the complete lack of amplification of the corresponding transcripts (ct ≥ 40 cycles) in 6/37 Caucasian and in 23/29 Chinese samples (see Fig. 4, panel a).

For the *UTS2*, we observed a significantly lower amplification of its transcripts in Caucasian blood samples in comparison to Chinese population; 29/33 Caucasian samples amplified > 38 cycles, while in Chinese population only 3/29 amplified so late (see Fig. 4, panel b). These results suggest minute number of *UTS2* transcripts in Caucasian blood samples, which is in accordance with results obtained from B-cell lines.

### Discriminating potential of two selected genes: *UTS2* and *UGT2B17*

To assess the population-discriminating potential of two identified transcripts: *UTS2* and *UGT2B17*, three different classifiers were built: binary decision trees (D.Tree), linear discriminant analysis (LDA) and support vector machines (SVM) with linear kernel. Classifiers were build based on Q_mean values derived from blood samples of both studied populations: Chinese (*n* = 29), and Caucasian (*n* = 37).

**Fig. 5:**
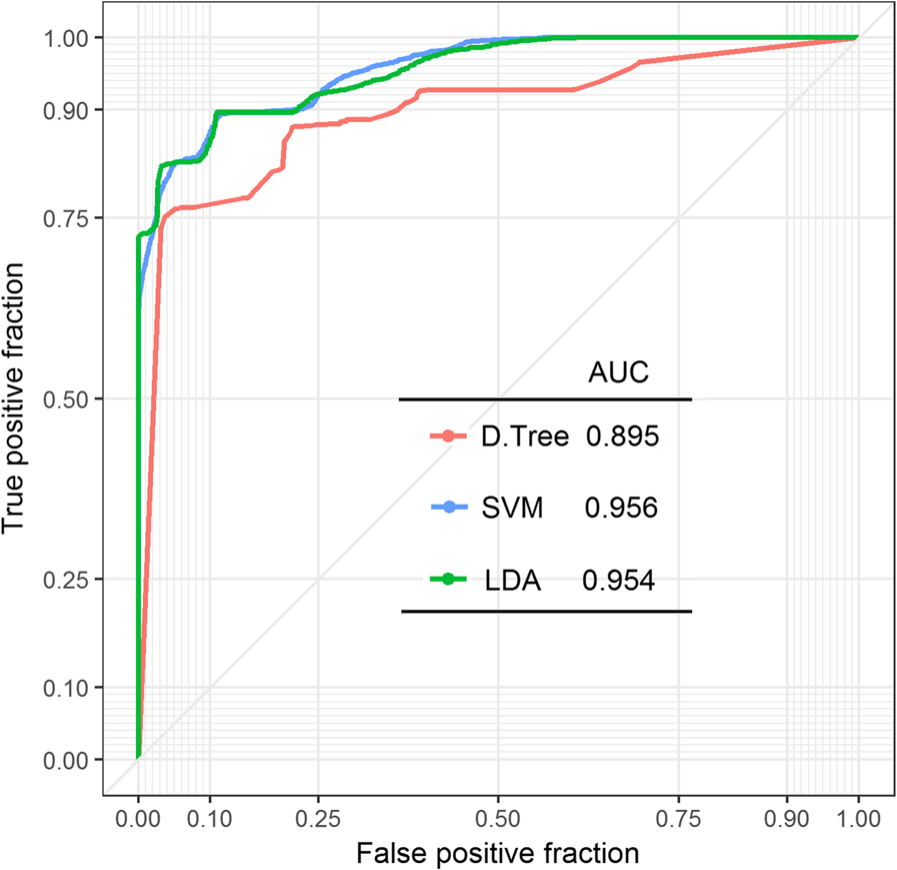
A ROC curve and AUC parameter calculated for 3 different classifiers: decision tree (D.Tree; red line), support vector machines (SVM; blue line), and linear discriminate analysis LDA (green line). Results were obtained based on blood samples collected from Chinese (*n* = 29) and Caucasian (*n* = 37) populations

The predictive ability of each classifier was assessed using 10-fold cross- validation, which was repeated 100 times due to moderate number of available cases. Classifiers were compared in terms of AUC (area under ROC curve) and F1 score (see Fig. 5, Additional file 1: Table S1 respectively). The analysis was conducted in R with the use of caret, e1071 and party libraries including plotROC and ggplot2 for visualization purposes.

The discriminative potential of all three tested classifiers can be assessed based on ROC curve shape and AUC parameter. The ROC curve was created by plotting the true positive fraction against the false positive fraction at various threshold settings. The shape of all presented curves follows the left-hand corner and the top border indicates the high accuracy of all 3 tested classifiers, of which SVM classifier can be considered as the most reliable one (AUC = 0.956 in Fig. 5). According to SVM classifier, the accuracy of sample assignment to one of the study population is close to 90%; 4/37 Caucasian samples have been incorrectly classified as Chinese population; whereas 3/29 Chinese samples were mistakenly ascribed to Caucasian population (see Additional file 2: Figure S1).

Regardless of classifier type our analysis indicates a high level of true positive results in comparison to false positive fraction (see the shape of curve and AUC parameter). Even the least accurate classification method (Decision Tree) gives highly sensitive results (~ 90%; see Additional file 1: Table S1). A scheme presenting discriminating potential of 2 genes (*UTS2* and *UGT2B17*) based on Decision Tree classifier is available in Additional file 3: Figure S2.

## Discussion

The aim of our study was to identify stable population-specific mRNA markers, representing the highest differences in gene expression between two human populations: Caucasian and Chinese. Only males were analyzed, to avoid gender-related differences in the expression level. Based on the high-throughput microarray analysis of B-lymphocyte cell lines representing Chinese and Caucasian populations, we have identified a set of 20 genes with the inter-population difference in the mean expression characterized by the at least 1.5-fold change and FDR < 0.05. The fold change of these 20 genes ranged from 1.5 to 2.5.

The validation of 13 transcripts from the 20 identified based on microarray study*,* for which specific TLDA probes were available, was performed on 47 independent cell lines. The differentiating status was confirmed for three genes: *UTS2*, *UGT2B17* and S*LC7A7*. The mean expression of *UTS2* was higher in CHB (25.8-fold change compared to CEU), while the expression of *UGT2B17* and *SLC7A7* was higher in CEU (3.2- and 2.2-fold change, respectively).

The magnitude of the population fold-change in *UGT2B17* or *UTS2* expression examined by dedicated TLDA cards was two to ten times higher than that revealed by during the whole-transcriptome screening by microarrays. Since this step of validation was performed in the same type of material (B-lymphocyte cell lines), these discrepancies were probably due to using different detection systems (microarrays, routinely used in transcriptome-wide screening experiments versus TLDA cards, targeting few preselected transcripts).

It is commonly known that lymphoblastoid cell lines (LCL) model is not perfect for gene expression studies, due to certain technical and environmental factors that may bias the results. The impact of Epstein Barr virus (EBV) transformation on the profile of gene expression in LCL is particularly important and widely discussed in the literature (e,g. [15–17]). It has been shown that a large number of genes are differently expressed between the primary and cultured cell lines; it has even been demonstrated that a subset of genes were expressed exclusively in EBV-transformed cells [17]. On the other hand, this effect is mostly important if the comparisons are made between the transformed and non-transformed cells; here, both populations analyzed by either microarrays or TLDA were represented by LCLs obtained from EBV transformed B-lymphocytes.

To exclude the possibility that the differences in the expression reflected specific conditions related to the maintenance of the CHB and CEU cell lines (for example bias in the sample collection time: CEU samples had been collected decades earlier than the CHB samples), the 2nd validation step was carried out using the primary biological material, i.e. peripheral blood samples obtained from Caucasian and Chinese males. Due to the limited availability of the blood samples, only two best-differentiating genes were subjected to this validation step. The inter-population differences in the expression was confirmed for both analyzed genes: the expression of *UTS2* was 13 times higher in Chinese (*p* **<** 0.001), while that of *UGT2B17* was six times higher in Caucasians (p **<** 0.001). The blood samples were neither subjected to EBV transformation nor to the collection time bias; we therefore believe, that the changes in *UGT2B17* and *UTS2* expression reflected true population-specific differences.

The discrepancies in the magnitude of the fold change between the first and the second step of validation require additional consideration. It could reflect differences in the expression between the homogeneous B-cell lines cultured under specific laboratory conditions and the peripheral blood samples composed of the mixture of different cells (B- and T-lymphocytes), whose expression might have been in addition affected by different environmental conditions of the donors.

On the other hand, some of the differences in the experiments using TLDA cards and qRT-PCR (which replaced the TLDA cards in the last phase of our study due to the budget restrictions) could cause probe-related differences in transcript detection. The first issue appears less important in the analysis of *UGT2B17* gene, which has only one transcript isoform. *UST2* gene however has three transcript isoforms; all were targeted by TaqMan probes, contrary to the qRT-PCR, where only two isoforms were covered. In addition, the TaqMan probe manufacturer (Life Technology database) has only recently announced that Hs00922170_m1 probe used in TLDA experiment might not be solely specific to *UTS2* transcripts.

The differences in the magnitude of the fold-changes notwithstanding, our results have confirmed that the population level of *UGT2B17* and *UTS2* expression differentiates Chinese and Caucasian populations, both in B-lymphocyte cell lines and in the whole peripheral blood samples. *UGT2B17* encodes a member of the uridine diphosphoglucuronosyltransferase protein family. The encoded enzyme takes a part in metabolism of steroids e.g. steroid hormones and lipid-soluble drugs (GeneCards). *UTS2* encodes a mature peptide that is an active cyclic heptapeptide and acts as a vasoconstrictor.

In the last step we performed a statistical analysis to confirm the discriminating power of the two genes (*UTS2 and UGT2B17*). Three different classifiers were built and after assessment of their sensitivity and specificity (ROC and AUC parameters), the sample population assignment was performed. In spite of the existing intra-population expression variation (see Figs. 2 and 4), our binary-classifiers showed high specificity (> 90%) and sensitivity (> 76%) in sample population classification. The accuracy of classification of an unknown sample to one of the studied populations was nearly 90% regardless of the classification method.

Gene expression differences among distinct human populations, especially in genes being under positive selection like *UGT2B17*, have been identified before [1, 3, 5, 9, 18]. These differences have been repeatedly shown to be heritable and linked to the variation across the human genome, potential mechanisms including INDELs or copy number variation (CNV), SNPs e.g. [2, 3, 5, 19] or alternative splicing [5, 9, 18]. Interestingly, we have noted that differences in the *UGT2B17* and *UTS2* expression in the studied groups were due to the complete lack of amplification in different number of individuals in both populations, rather than to the subtle population-specific fluctuations in the expression level. These observations suggested that the individuals, where no transcription of a given gene was observed (ct ≥ 40), could be homozygotes for an expression-abolishing mutation.

To shed light on the mechanisms underlying the population differences in the level of *UGT2B17* and *UTS2* expression, we examined SNPs with population-specific allele frequencies listed in the genome databases as well as our earlier data from Infinium Human OmniExpressExome, obtained for the same cell lines as used here in the discovery phase (see Additional file 4) and [20]. No SNPs were found, which would affect expression of *UGT2B17* and *UTS2* genes in the 0–1 manner (e.g. causing premature termination codons or obvious spice site alterations).

Twenty-five SNPs, which correlated with population differences in *UTS2* gene expression (see Additional file 5: Table S2), were located far away (from 280,000 to 360,000 bp up- and from 52,000 bp- 920,000 bp down-) from the gene. Further studies are required to investigate whether these SNPs have an impact on the regulation of *UTS2* expression. For *UGT2B17*, no correlation between population differences in gene expression and SNPs was identified.

Another mechanism that may play a role in the regulation of gene expression is methylation of DNA; e.g. methylation of the gene promoter region leads to gene expression silencing. Examination of our earlier data obtained from Illumina Infinium Human Methylation 450 BeadChip Microarray for the same set of Chinese and Caucasian cell lines clearly indicated the lack of methylation differences that would affect the level of gene expression in *UTS2* and *UGT2B17* genes ( [21] and data not published).

Interestingly, it has been shown that *UGT2B17* gene lies in the genomic region where numerous CNV (copy number variants) occur (see ENSEMBL database, and e.g. [7, 22–24]. Some of them, e.g. *esv3600874, esv3600873, esv3600875*, are characterized by high inter-population variation in allele frequency, and *UGT2B17* deletion alleles are more common in East Asians, than in Africans and Europeans (e.g. [22, 24, 25]). Our results, where the complete lack of *UGT2B17* amplification was more frequent among Chinese compared to Caucasian cell lines (56 to 23%), are in accord with the scenario of CNV deletion underlying the lower *UGT2B17* expression in Chinese group. In fact, the majority of the cell lines, where *UGT2B17* transcripts were not amplified in TLDA cards are listed in the ENSEMBL database as carrying *esv3600874*, *esv3600873*, *esv3600875* deletions encompassing the whole gene or its large part. Although no genotype information (i.e. information whether the individual has a hetero- vs homozygous deletion) is available in that database, it is highly probable, that in the samples, which did not amplify in our settings, the deletion was present on both alleles.

Based on the ENSEMBL database, *UTS2* also lies in the region rich in CNV polymorphisms. However, the only reported CNV (*esv3585131*) exhibiting inter-population difference in the allele frequency lies in the long intron 1. The possibility that, similarly to *UGT2B17*, this CNV affects the expression profile, is therefore not strong, although the possibility that it may influence splicing and affect the gene expression regulation cannot be excluded. Some genomic studies have identified CNVs lying at the larger distance from *UTS2*, but so far there is no proof for their role in the *UTS2* expression regulation e.g. [23]. Another explanation may involve the so called novel transcribed regions. Based on the transcriptome sequencing of Chinese and Caucasian population samples, over 1600 putative ethnic-specific novel transcribed regions that may influence gene expression have been recently identified [19]; importantly, *UTS2* gene was among 20 genes reported to exhibit population-specific gene expression pattern and at the same time to encompass novel transcribed regions in Chinese population [26].

## Conclusions

The classification accuracy of our binary classifiers, which seems reasonably high for the limited number of samples examined, may either decrease or increase in a larger-sample-number study. For a conclusive corroboration, further studies encompassing larger population groups need to be carried out. Nonetheless, our study provides a preliminary evidence that changes in gene expression between human populations may be treated as a potential population marker applicable for human population identification.

Our findings may have a practical application in the field of forensic science. In particular, the differentiating transcripts, targeted by the specific probes, may be used as population-specific markers in the efforts aiming to separate mixture of blood from individuals of different populations. In fact, our preliminary study performed on *UGT2B17* transcript labeled with the FISH probes and LCM technology, showed that a mixture of B-cell lines from Caucasian and Chinese population could be separated (data not shown). However, further studies are necessary to confirm these results on other mRNA transcripts and on a different biological material.

## Methods

### RNA samples

RNA samples from unrelated males representing Caucasians and Chinese populations (further referred to as CEU and CHB, respectively), were isolated either from B-lymphocyte cell lines (Coriell Cell Repositories) or from the samples of peripheral blood (for details see Fig. 1 and Additional file 6: Table S3).

Both B-lymphocyte cell lines and peripheral blood samples underwent identical procedures including: RNA isolation (RNeasy Mini kit, Qiagen), evaluation of its purity and integrity (RIN) and reverse transcription into cDNA (RNA isolation procedures in Additional file 7). RNA quality and quantity was determined in Agilent 2100 Bioanalyzer (Agilent Technologies). RNA samples characterized by RIN values in the range 8–9.5 were reversely transcribed into cDNA by using the Enhanced Avian RT First Strand Synthesis Kit (Sigma).

### Study design

The study consisted of three main phases: a discovery and a two-step validation (Fig. 1).

### Discovery phase: Microarray analysis of the transcripts from B-lymphocyte cell lines

B-lymphocyte cell lines from CEU (*n* = 35) and CHB (*n* = 32) were examined on HumanHT-12v4 Expression BeadChip Kit expression arrays (Illumina), according to the manufacturer-specified procedure. Technical quality evaluation (signal intensity, background level, noise level, the number of detected actively expressed genes, or hybridization control of hybridized RNA samples) was performed in Genome Studio V2010.1 program. Then, an unsupervised analysis was performed to eliminate any technical factors that may influence measurement reliability. Lastly, to select genes exhibiting differences in the gene expression level between two studied populations, the supervised analysis was performed using the Student’s *t* test (Detailed information in Additional file 8).

The set of differentially expressed genes (Table 1), satisfying the threshold of 1.5 fold difference, *p* < 0.05 and false discovery ratio FRD < 0.05, was selected and was subjected to a two-step validation procedure.

### Validation phase

#### Step 1: Validation of the selected transcripts in independent B-cell lines

The population-differentiating transcripts were validated on B-lymphocyte cell lines using TaqMan Low Density Arrays (TLDA) (ThermoFisher Scientific) (Detailed information regarding TLDA experiment in Additional file 9). The validation was performed in 47 independent B-lymphocyte cell lines from both studied populations (CEU: *n* = 22; CHB: *n* = 25). Ten of the cell lines used in the Discovery phase were additionally analyzed for the technical testing of microarray results (see Additional file 6: Table S3). Only 13 out of 20 population-differentiating transcripts were examined; their selection was based on i) TaqMan probes availability in ThermoFisher Scientific probe database, and ii) probes specificity towards the examined transcripts. Three the most stable housekeeping genes (*GAPDH, IPO8, PPIA*) were selected to normalize the experiments, based on the preliminary test performed using Housekeeping TLDA cards (ThermoFisher Scientific) (for a list of mRNA transcripts and TLDA probes see: Additional file 10: Table S4). A mixture of five CEU samples was used as a calibrator. Amplification curves were analyzed with RQ Manager Software (ThermoFisher Scientific).

The normality of the distribution was analyzed with the Shapiro- Wilk test.

Analysis of the fold-change and *p*-values characterizing the differences in the mean level of amplification of the selected transcripts in both populations was performed with Data Assist software (ThermoFisher Scientific) and presented in Table 2. The significance of differences in the expression between both studied populations was tested using Mann-Whitney U test, performed with Statistica v.9.0. software.

#### Step 2: Validation of the confirmed population-differentiating transcripts in the peripheral blood samples

The population-differentiating status of the transcripts characterized by the fold change > 3 in the 1st step of validation was further analyzed in the material isolated from peripheral blood samples (CEU: *n* = 37 and CHB: *n* = 29), using7900 HT Fast Real-Time PCR system (Life Technologies, Carlsbad, CA, USA) with Universal Probe Library fluorescence probes (Roche, Basel, Switzerland) and 5′- nuclease assay. The results were normalized according to the previously described model [27], using GeNorm application [28] with a combination of three housekeeping genes: *EIF3S10, UBE2D2, HADHA*. The significance of differences in the expression between both studied populations was tested using Mann-Whitney U test in Statistica v.9.0. software.

The details allowing identification of the probes and primers used in 2nd validation steps are available in Additional file 11: Table S5.

## Additional files

Additional file 1 : **Table S1**. A results of 3 classifiers cross-validation. (DOCX 13 kb)

Additional file 2 : **Figure S1**. The location of optimal hyperplane (black line) and supporting vectors (yellow lines) determined based on SVM method. (DOCX 42 kb)

Additional file 3 : **Figure S2**. A binary Decision-Tree classifier built based on UTS2 and UGT2B17 data (left Panel) and for UTS2 (Right Panel) obtained from Caucasian (*n* = 37), and Chinese (*n* = 29) blood samples. (DOCX 87 kb)

Additional file 4 : Infinium Human OmniExpressExome microarray. (DOCX 11 kb)

Additional file 5 : **Table S2**. Correlation between SNPs and gene expression for *UTS2* gene. No such correlation was identified for *UGT2B17* gene. (DOCX 14 kb)

Additional file 6 : **Table S3**. A list of B-cell lines used in Microarray analysis and TLDA experiment. (DOCX 21 kb)

Additional file 7 : RNA isolation procedure. (DOCX 10 kb)

Additional file 8 : Microarray analysis. A detailed description of Microarray statistical analysis. (DOCX 33 kb)

Additional file 9 : TLDA experiment. A detailed description of the experiment carried out on TLDA cards. (DOCX 11 kb)

Additional file 10 : **Table S4**. List of mRNA transcripts and TLDA probes. (DOCX 14 kb)

Additional file 11 : **Table S5**. Primer design for qRT-PCR validation experiment. (DOCX 13 kb)

## Abbreviations

AUC: Area under the curve
CEU: Caucasian population
CHB: Chinese population
LCL: Lymphoblastoid cell lines
LDA: Linear discriminant analysis
ROC: a receiver operating characteristic curve
SVM: Support vector machines
TLDA: TaqMan low density arrays
