# Supplementary material for: Transcriptomic population markers for human population discrimination
Source: BMC Genet. 2018 Aug 7;19:54. doi: 10.1186/s12863-018-0663-2 (PMC6081795; doi:10.1186/s12863-018-0663-2)
Supplement: Supplementary file 1 — : Table S1. A results of 3 classifiers cross-validation. (DOCX 13 kb) [file 12863_2018_663_MOESM1_ESM.docx]

**Additional file 1: Table S1.** A results of 3 classifiers cross-validation.

| **Classifier type** | **Features** | **Accuracy** | **Precision** | **Sensitivity** | **Specificity** | **F1** | **AUC** |
| --- | --- | --- | --- | --- | --- | --- | --- |
| ctree | UTS2/UGT2B17 | 0.89 | 1.00 | 0.76 | 1.00 | 0.863 | 0.895 |
| SVMLinear | UTS2/UGT2B17 | 0.89 | 0.92 | 0.83 | 0.95 | 0.873 | 0.956 |
| LDA | UTS2/UGT2B17 | 0.89 | 0.89 | 0.83 | 0.92 | 0.857 | 0.954 |
