# Supplementary material for: Transcriptomic population markers for human population discrimination
Source: BMC Genet. 2018 Aug 7;19:54. doi: 10.1186/s12863-018-0663-2 (PMC6081795; doi:10.1186/s12863-018-0663-2)
Supplement: Supplementary file 2 — : Figure S1. The location of optimal hyperplane (black line) and supporting vectors (yellow lines) determined based on SVM method. (DOCX 42 kb) [file 12863_2018_663_MOESM2_ESM.docx]

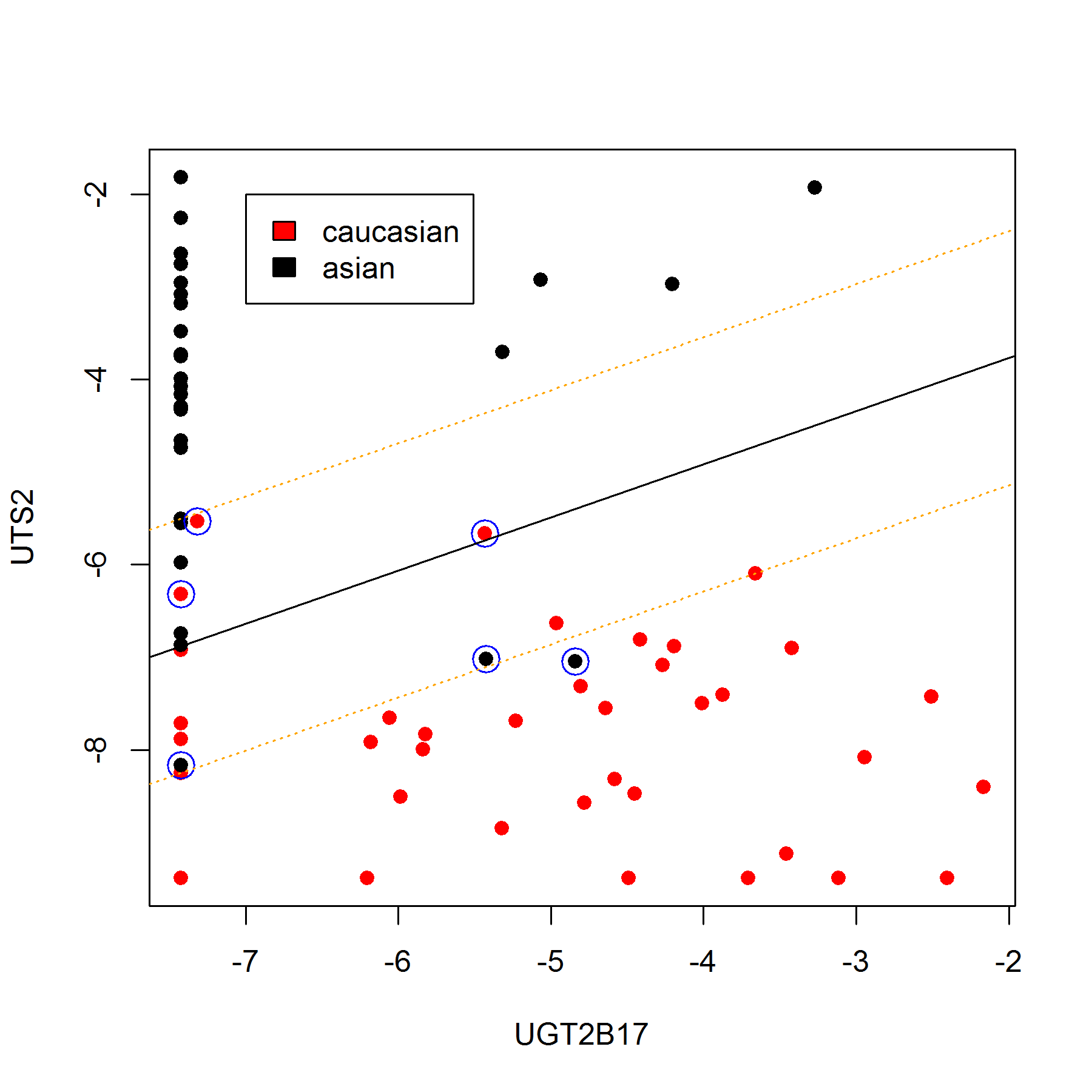


**Additional file 2: Figure S1.** The location of optimal hyperplane (black line) and supporting vectors (yellow lines) determined based on SVM method. Caucasian samples are marked with red, Chinese with black dots. Samples incorrectly assigned to population are marked with blue circles.
