# Supplementary material for: Transcriptomic population markers for human population discrimination
Source: BMC Genet. 2018 Aug 7;19:54. doi: 10.1186/s12863-018-0663-2 (PMC6081795; doi:10.1186/s12863-018-0663-2)
Supplement: Supplementary file 3 — : Figure S2. A binary Decision-Tree classifier built based on UTS2 and UGT2B17 data (left Panel) and for UTS2 (Right Panel) obtained from Caucasian (n = 37), and Chinese (n = 29) blood samples. (DOCX 87 kb) [file 12863_2018_663_MOESM3_ESM.docx]

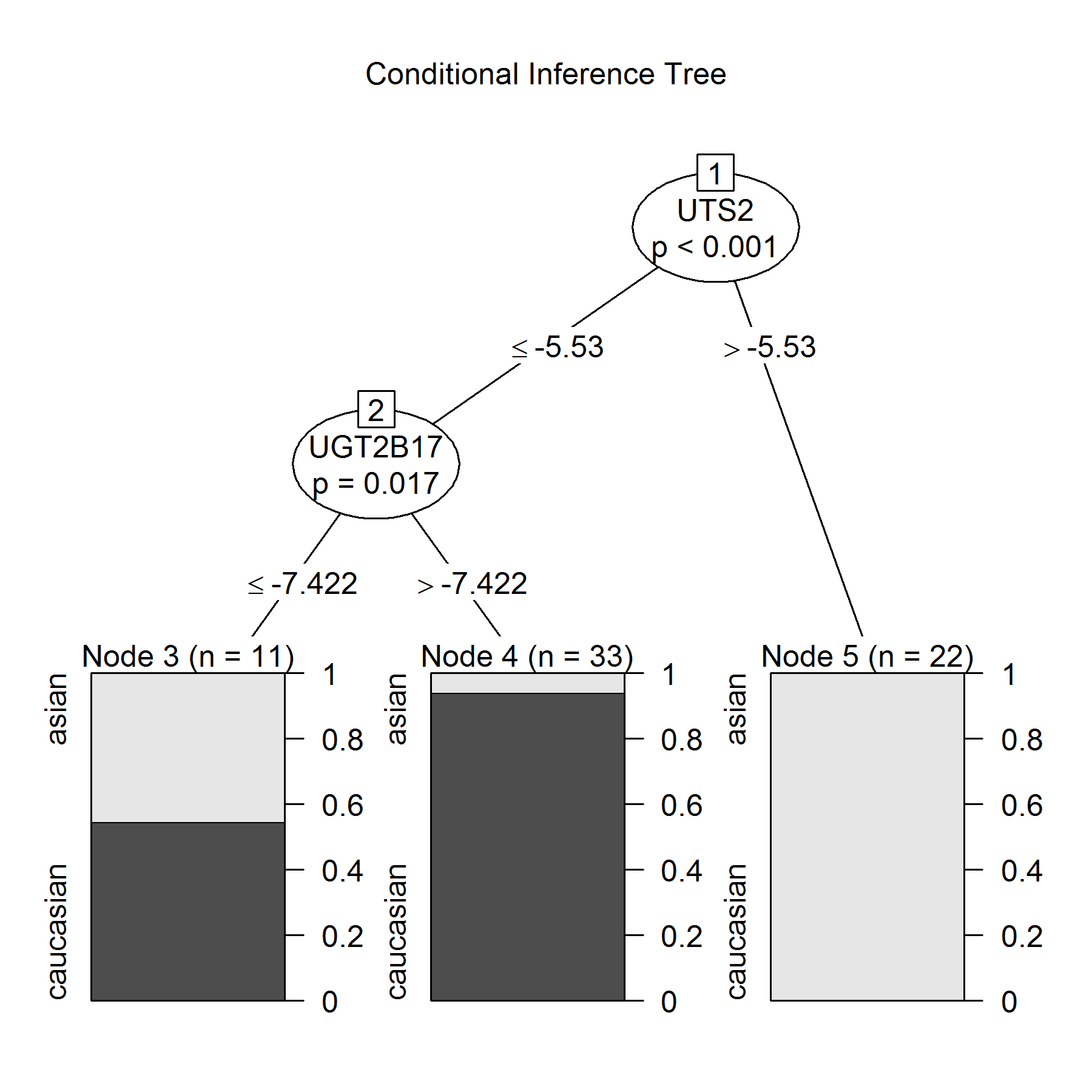

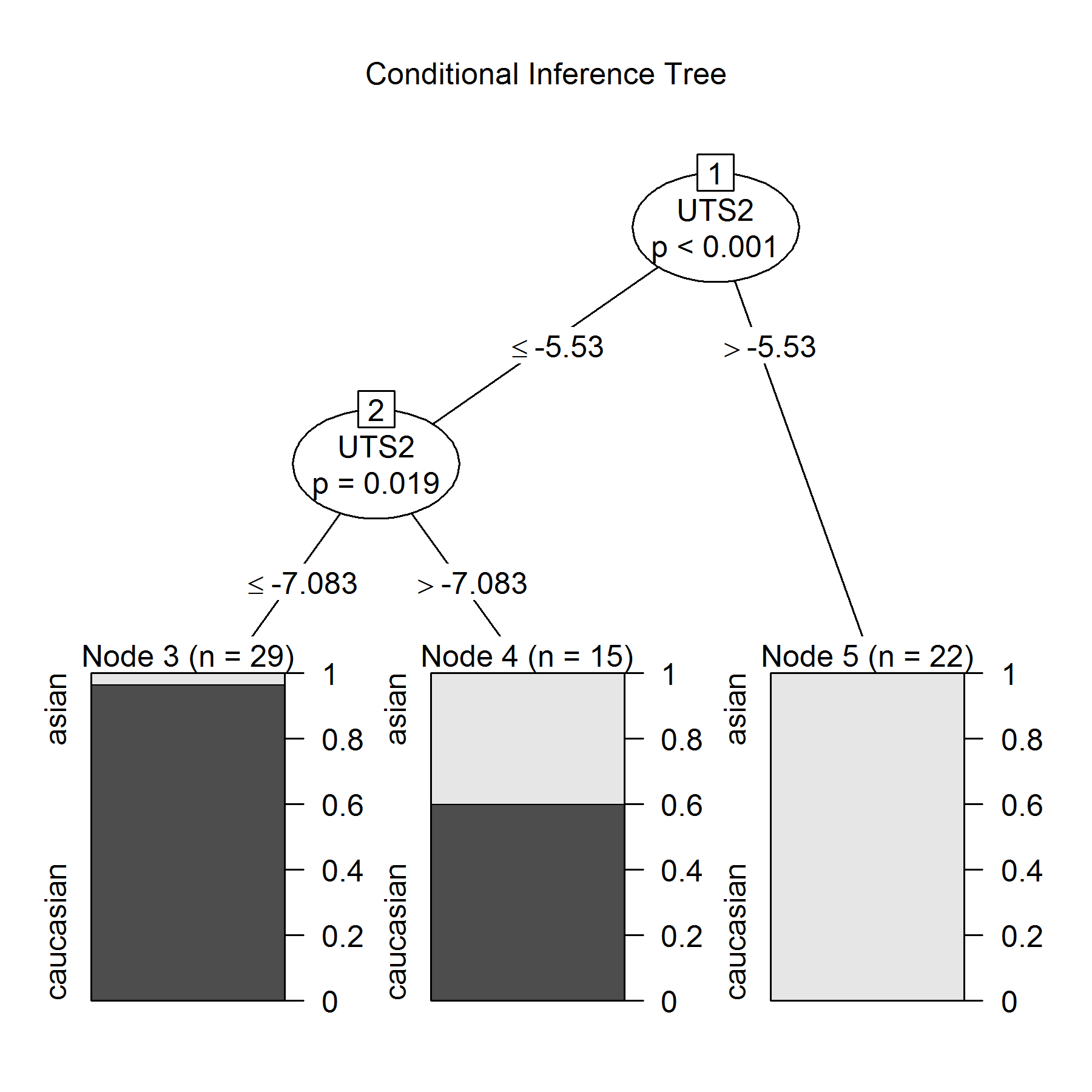


**Additional file 3: Figure S2.** A binary Decision-Tree classifier built based on UTS2 and UGT2B17 data (left Panel) and for UTS2 (Right Panel) obtained from Caucasian (n=37), and Chinese (n= 29) blood samples. Caucasian population denotes black color; Chinese population is presented in bright color.
