# Supplementary material for: Transcriptomic population markers for human population discrimination
Source: BMC Genet. 2018 Aug 7;19:54. doi: 10.1186/s12863-018-0663-2 (PMC6081795; doi:10.1186/s12863-018-0663-2)
Supplement: Supplementary file 4 — : Infinium Human OmniExpressExome microarray. (DOCX 11 kb) [file 12863_2018_663_MOESM4_ESM.docx]

**Additional file 4.** Infinium Human OmniExpressExome microarray

The analysis of correlation between gene expression and SNPs was performed based on data obtained from HumanHT-12v4 Expression BeadChip Kit expression arrays and Infinium HumanOmniExpressExome microarrays (for methodology see reference (20) in the main text).The same set of 32 B-lymphocyte cell lines from CEU and CHB populations was examined on both microarrays.

After initial data filtering, all SNPs with minor allele frequency <0.05 were rejected from further analysis. Subsequently, for both tested genes (*UTS2* and *UGT2B17*) all SNPs located within 1 mln bp up- and down- from the Illumina gene expression probes were analyzed. To assess the relation between the level of gene expression and the SNP genotype, a variance analysis (ANOVA) was performed.

For each probe, a statistical significance (p-value) was determined, and the adjusted value was calculated using the correction for multiple False Discovery Rate (FDR). For 25 SNPs, correlation with gene expression (FDR< 0.05) was identified (see **Additional file 5: Tab S2**).
