# Supplementary material for: Transcriptomic population markers for human population discrimination
Source: BMC Genet. 2018 Aug 7;19:54. doi: 10.1186/s12863-018-0663-2 (PMC6081795; doi:10.1186/s12863-018-0663-2)
Supplement: Supplementary file 5 — : Table S2. Correlation between SNPs and gene expression for UTS2 gene. No such correlation was identified for UGT2B17 gene. (DOCX 14 kb) [file 12863_2018_663_MOESM5_ESM.docx]

**Additional file 5: Table S2.** Correlation between SNPs and gene expression for *UTS2* gene. No such correlation was identified for *UGT2B17* gene.

| **Gene name** | **SNP** | **ANOVA**  **p-value** | **ANOVA**  **FDR** |
| --- | --- | --- | --- |
| UTS2 | rs7526892 | 8.92E-005 | 3.99E-02 |
| UTS2 | rs1725262 | 3.53E-006 | 1.63E-03 |
| UTS2 | rs170551 | 2.27E-006 | 1.05E-03 |
| UTS2 | rs12068202 | 2.44E-011 | 1.16E-08 |
| UTS2 | rs161803 | 3.68E-008 | 1.74E-05 |
| UTS2 | exm2264984 | 3.68E-008 | 1.74E-05 |
| UTS2 | rs161827 | 3.68E-008 | 1.74E-05 |
| UTS2 | rs161811 | 3.68E-008 | 1.74E-05 |
| UTS2 | rs161810 | 3.68E-008 | 1.74E-05 |
| UTS2 | rs519546 | 3.68E-008 | 1.74E-05 |
| UTS2 | rs150914 | 1.91E-008 | 9.06E-06 |
| UTS2 | rs406809 | 7.40E-008 | 3.46E-05 |
| UTS2 | rs161802 | 4.42E-005 | 2.00E-02 |
| UTS2 | rs225121 | 2.95E-008 | 1.40E-05 |
| UTS2 | rs397349 | 4.42E-005 | 2.00E-02 |
| UTS2 | rs7537362 | 5.16E-006 | 2.37E-03 |
| UTS2 | rs7523335 | 3.43E-006 | 1.58E-03 |
| UTS2 | rs6577466 | 1.26E-006 | 5.86E-04 |
| UTS2 | rs1542387 | 4.70E-005 | 2.11E-02 |
| UTS2 | rs10746477 | 4.44E-006 | 2.04E-03 |
| UTS2 | rs7520373 | 2.50E-005 | 1.13E-02 |
| UTS2 | rs12045438 | 1.96E-005 | 8.91E-03 |
| UTS2 | rs7545384 | 2.39E-005 | 1.09E-02 |
| UTS2 | exm-rs12025126 | 1.14E-006 | 5.31E-04 |
| UTS2 | rs10864363 | 6.75E-007 | 3.15E-04 |
