# Supplementary material for: Transcriptomic population markers for human population discrimination
Source: BMC Genet. 2018 Aug 7;19:54. doi: 10.1186/s12863-018-0663-2 (PMC6081795; doi:10.1186/s12863-018-0663-2)
Supplement: Supplementary file 6 — : Table S3. A list of B-cell lines used in Microarray analysis and TLDA experiment. (DOCX 21 kb) [file 12863_2018_663_MOESM6_ESM.docx]

**Additional file 6: Table S3.** A list of B-cell lines used in Microarray analysis and TLDA expression study.

| nb | sample | population id | study |
| --- | --- | --- | --- |
| 1 | AG 19496 | CEU | Microarray |
| 2 | AG 19540 | CEU | Microarray |
| 3 | AG 18271 | CEU | Microarray |
| 4 | AG 18257 | CEU | Microarray |
| 5 | AG 18244 | CEU | Microarray |
| 6 | AG 18300 | CEU | Microarray |
| 7 | AG 18302 | CEU | Microarray |
| 8 | AG 19409 | CEU | Microarray |
| 9 | AG 19412 | CEU | Microarray |
| 10 | AG 19414 | CEU | Microarray |
| 11 | AG 19580 | CEU | Microarray/TLDA |
| 12 | AG 18224 | CEU | Microarray |
| 13 | AG 18252 | CEU | Microarray |
| 14 | AG 19416 | CEU | Microarray |
| 15 | AG 19587 | CEU | Microarray/TLDA |
| 16 | AG 18276 | CEU | Microarray/TLDA |
| 17 | AG 19579 | CEU | Microarray/TLDA |
| 18 | AG 19505 | CEU | Microarray |
| 19 | AG 18250 | CEU | Microarray |
| 20 | AG 18236 | CEU | Microarray |
| 21 | AG 18219 | CEU | Microarray |
| 22 | AG 18183 | CEU | Microarray |
| 23 | AG 18193 | CEU | Microarray/TLDA |
| 24 | AG 19623 | CEU | Microarray |
| 25 | AG 18216 | CEU | Microarray |
| 26 | AG 18189 | CEU | Microarray |
| 27 | AG 19411 | CEU | Microarray |
| 28 | AG 19524 | CEU | Microarray |
| 29 | AG 18228 | CEU | Microarray |
| 30 | AG 19497 | CEU | Microarray |
| 31 | AG 19506 | CEU | Microarray |
| 32 | AG 19406 | CEU | Microarray |
| 33 | AG 19520 | CEU | Microarray |
| 34 | AG 19534 | CEU | Microarray |
| 35 | AG 19495 | CEU | Microarray |
| 36 | GM 18524 | CHB | Microarray |
| 37 | GM 18534 | CHB | Microarray |
| 38 | GM 18536 | CHB | Microarray |
| 39 | GM 18544 | CHB | Microarray |
| 40 | GM 18557 | CHB | Microarray |
| 41 | GM 18558 | CHB | Microarray |
| 42 | GM 18561 | CHB | Microarray |
| 43 | GM 18562 | CHB | Microarray |
| 44 | GM 18563 | CHB | Microarray |
| 45 | GM 18572 | CHB | Microarray |
| 46 | GM 18603 | CHB | Microarray |
| 47 | GM 18605 | CHB | Microarray |
| 48 | GM 18606 | CHB | Microarray |
| 49 | GM 18608 | CHB | Microarray/TLDA |
| 50 | GM 18609 | CHB | Microarray/TLDA |
| 51 | GM 18611 | CHB | Microarray/TLDA |
| 52 | GM 18612 | CHB | Microarray |
| 53 | GM 18621 | CHB | Microarray |
| 54 | GM 18622 | CHB | Microarray |
| 55 | GM 18623 | CHB | Microarray |
| 56 | GM 18624 | CHB | Microarray |
| 57 | GM 18632 | CHB | Microarray |
| 58 | GM 18633 | CHB | Microarray/TLDA |
| 59 | GM 18635 | CHB | Microarray |
| 60 | GM 18636 | CHB | Microarray/TLDA |
| 61 | GM 18637 | CHB | Microarray |
| 62 | GM 18559 | CHB | Microarray |
| 63 | GM 18639 | CHB | Microarray |
| 64 | GM 18643 | CHB | Microarray |
| 65 | GM 18647 | CHB | Microarray |
| 66 | GM 18638 | CHB | Microarray |
| 67 | GM 18740 | CHB | Microarray |
| 68 | AG 15200 | CEU | TLDA |
| 69 | AG 15202 | CEU | TLDA |
| 70 | AG 15205 | CEU | TLDA |
| 71 | AG 15206 | CEU | TLDA |
| 72 | AG 15208 | CEU | TLDA |
| 73 | AG 17003 | CEU | TLDA |
| 74 | AG 17008 | CEU | TLDA |
| 75 | AG 17007 | CEU | TLDA |
| 76 | AG 17010 | CEU | TLDA |
| 77 | AG 17324 | CEU | TLDA |
| 78 | AG 17325 | CEU | TLDA |
| 79 | AG 17326 | CEU | TLDA |
| 80 | AG 18190 | CEU | TLDA |
| 81 | AG 18192 | CEU | TLDA |
| 82 | AG 18245 | CEU | TLDA |
| 83 | AG 18254 | CEU | TLDA |
| 84 | AG 18305 | CEU | TLDA |
| 85 | CZ-AG15724 | CEU | TLDA |
| 86 | CZ-AG 15725 | CEU | TLDA |
| 87 | CZ-AG 15726 | CEU | TLDA |
| 88 | CZ-AG15727 | CEU | TLDA |
| 89 | CZ-AG15728 | CEU | TLDA |
| 90 | GM 17965 | CHB | TLDA |
| 91 | GM 17967 | CHB | TLDA |
| 92 | GM 17969 | CHB | TLDA |
| 93 | GM 17972 | CHB | TLDA |
| 94 | GM 17973 | CHB | TLDA |
| 95 | GM 17974 | CHB | TLDA |
| 96 | GM 17975 | CHB | TLDA |
| 97 | GM 17976 | CHB | TLDA |
| 98 | GM 17979 | CHB | TLDA |
| 99 | GM 17980 | CHB | TLDA |
| 100 | GM 17983 | CHB | TLDA |
| 101 | GM 18530 | CHB | TLDA |
| 102 | GM 18543 | CHB | TLDA |
| 103 | GM 18546 | CHB | TLDA |
| 104 | GM 185548 | CHB | TLDA |
| 105 | GM 18549 | CHB | TLDA |
| 106 | GM 18613 | CHB | TLDA |
| 107 | GM 18620 | CHB | TLDA |
| 108 | GM 18645 | CHB | TLDA |
| 109 | GM 18745 | CHB | TLDA |
| 110 | J-GM 18940 | CHB | TLDA |
| 111 | J-GM 18945 | CHB | TLDA |
| 112 | J-GM 18948 | CHB | TLDA |
| 113 | J-GM 18953 | CHB | TLDA |
| 114 | J-GM 18959 | CHB | TLDA |
