# Supplementary material for: Transcriptomic population markers for human population discrimination
Source: BMC Genet. 2018 Aug 7;19:54. doi: 10.1186/s12863-018-0663-2 (PMC6081795; doi:10.1186/s12863-018-0663-2)
Supplement: Supplementary file 7 — : RNA isolation procedure. (DOCX 10 kb) [file 12863_2018_663_MOESM7_ESM.docx]

**Additional file 7.** RNA isolation procedure

Total RNA was isolated from 80 cell lines with the use of RNeasy Mini Kit and RNeasy Plus Mini Kit (Qiagen). RNA isolates corresponding to the cell lines that have not been previously subjected to microarray analysis were selected for TaqMan Array analysis. Nucleic acid concentration was assessed by spectrophotometric analysis, i.e., UV absorbance measurement at 260 nm (A260) using the NanoDrop ND-1000 apparatus. RNA quality was determined by capillary electrophoresis in Agilent 2100 Bioanalyser, by applying the RNA 6000 Nano Assay (Agilent Technologies) and the suitable RNA 6000 Nano Marker. RNA integrity was determined by applying the RNA Integrity Number (RIN). The obtained RNA isolates were of good quality as confirmed by the observed RIN values in the range 8–9.5, and fulfilled the quality criteria for further analyses. Aliquots of RNAs obtained from 72 cell lines were reversely transcribed into cDNA by using the Enhanced Avian RT First Strand Synthesis Kit (Sigma).
