# Supplementary material for: Transcriptomic population markers for human population discrimination
Source: BMC Genet. 2018 Aug 7;19:54. doi: 10.1186/s12863-018-0663-2 (PMC6081795; doi:10.1186/s12863-018-0663-2)
Supplement: Supplementary file 8 — : Microarray analysis. A detailed description of Microarray statistical analysis. (DOCX 33 kb) [file 12863_2018_663_MOESM8_ESM.docx]

**Additional file 8.** Microarray analysis

Unsupervised analysis

Unsupervised analysis was performed by means of hierarchical clustering, the results of which are usually presented in a dendrogram. The hierarchical clustering (in each step of the analysis) consists of the calculation of the matrix of distances between all objects, the creation of clusters by combining the objects and/or clusters created in the previous step, and the calculation of the distances between clusters combining several objects. In the clustering the Euclidean distance was used to calculate the distances between samples, while the *Ward’s* method was applied to combining individual clusters.

The aim of this step of the analysis was to detect and eliminate any technical factors and to filter out, based on biological replicates of cellular lines, the genes displaying high measurement reliability.

The clustering performed for all the probes on the microarray revealed as the main source of variability in the analyzed dataset a variability between individual microarrays.

In order to eliminate the effect of individual microarrays on the obtained profiles of gene expression, the so called data centering method was applied (i.e. bringing the mean expression of each gene within one microarray to a common value). This step is performed as follows:

- for each gene, the mean expression in particular arrays is calculated by the following formula:

-for each gene and each sample, the mean value of the gene obtained in the array, on which a given sample was hybridized is subtracted:

Based on the "centered" probes, a hierarchical clustering was repeated, which in result proved the effectiveness of the applied method in eliminating the effects of individual microarrays.

Filtering genes displaying high measurement reliability

Despite the elimination of the effect of individual microarrays, a small number of samples, for which biological replicates were created, did not group together, which implied insufficient reliability of the expression measurement in case of a certain, significant in numbers, group of genes. Therefore a novel method of filtering out genes with the high reliability of measurement (“measurable” genes) was developed, that followed the procedure below:

-for each gene, based on all samples, the coefficient of variation was calculated:

, where *ĝ_i_* - mean expression of the *i* gene

-for each gene the coefficient of variation was calculated for each line with three biological repetitions:

where *j*=1,2,…,23- lines that have three repetitions

*k*=1,2,3 -*k*. repetition of the *j* line

*ĝ_ijk –_* mean expression of the *i* gene in the *j* line

-for each gene the third quartile of the coefficient of variation was calculated:

*vc.3q_i_ = centile((vc_i1_, vc_i2_, …, vc_i23_), 0.75)*

-for each gene the measurability coefficient was calculated as the quotient of the total variation and the third quartile of the variation coefficient of this gene :

In an arbitrary decision, the genes deemed as “measurable” were those, for which “the coefficient of measurability" *mc >* 1.5. A total of 3732 genes were filtered out as fulfilling this criterion. The hierarchical clustering of the analyzed samples based on the “measurable” genes showed an ideal grouping of all available biological replicates.

Supervised analysis - selection of genes discriminating between the Caucasian and Asian populations.

A supervised analysis was carried out using the Student's *t* test for independent samples. A total of 67 male cell lines were analyzed, with 35 males from the Caucasian and 32 from the Asian population. The test was performed for 3732 probes that met the measurability criterion *mc >* 1.5. For each probe, a statistical significance (p-value) and the adjusted value were determined by applying the correction for multiple False Discovery Rate (FDR) testing and for the fold-change in expression level within the analyzed populations.

As a result of the supervised analysis, 189 genes were selected that met the FDR<5% criterion.
