# Supplementary material for: Transcriptomic population markers for human population discrimination
Source: BMC Genet. 2018 Aug 7;19:54. doi: 10.1186/s12863-018-0663-2 (PMC6081795; doi:10.1186/s12863-018-0663-2)
Supplement: Supplementary file 9 — : TLDA experiment. A detailed description of the experiment carried out on TLDA cards. (DOCX 11 kb) [file 12863_2018_663_MOESM9_ESM.docx]

**Additional file 9.** TLDA experiment

In the first step of validation, in order to select the reference genes stably expressed in the examined system, a test reaction was carried out using the TLDA card (ThermoFisher Scientific) containing 16 HKG (housekeeping) genes typically used in gene expression studies. Based on the above experiment, the three most stable genes: *GAPDH*, *IPO8* and *PPIA* were selected for further analyses.

Validation with the use of the 384-well TLDA cards involved 13 genes selected based on the microarray analysis, for which the probe sets were selected from the ThermoFisher Scientific database, so to identify the same transcripts as those detected by the probes used in microarray analysis. The measurement of relative gene expression using the TLDA cards was conducted in three repetitions for each of the genes tested. The wells of the 384-well TLDA cards were loaded with 100ul aliquots of the reaction mix containing cDNA, Gene Expression Master mix (ThermoFisher Scientific) and water. Next, the TLDA cards were centrifuged twice at 1200rpm for 1min in Heraeus centrifuge (Thermo Scientific), and sealed with a card sealer to prevent well-to-well contamination. Finally, the TLDA cards containing 8 analyzed samples and microarray classifier genes were processed using the Real-Time PCR 7900 HT system (ThermoFisher Scientific).
