# Supplementary material for: Transcriptomic population markers for human population discrimination
Source: BMC Genet. 2018 Aug 7;19:54. doi: 10.1186/s12863-018-0663-2 (PMC6081795; doi:10.1186/s12863-018-0663-2)
Supplement: Supplementary file 10 — : Table S4. List of mRNA transcripts and TLDA probes. (DOCX 14 kb) [file 12863_2018_663_MOESM10_ESM.docx]

**Additional file 10: Table S4.** List of mRNA transcripts and TLDA probes

| **mRNA transcript name** | **TLDA probe name** | **Type of probe** |
| --- | --- | --- |
| C1orf115 | Hs00226770_m1 | Target |
| CDC42EP5 | Hs00414771_m1 | Target |
| CHI3L2 | Hs00970220_m1 | Target |
| CYP1B1 | Hs00164383_m1 | Target |
| IFITM3 | Hs03057129_s1 | Target |
| MOXD1 | Hs01026921_m1 | Target |
| PLA2G4C | Hs01003743_m1 | Target |
| S1PR4 | Hs02330084_s1 | Target |
| SLC7A7 | Hs00909952_m1 | Target |
| TBC1D4 | Hs00207999_m1 | Target |
| UGT2B17 | Hs00854486_sH | Target |
| UGT2B7 | Hs00426592_m1 | Target |
| UTS2 | Hs00922170_m1 | Target |
| *GAPDH* | *Hs99999905_m1* | *HKG-1* |
| *IPO8* | *Hs00183533_m1* | *HKG-2* |
| *PPIA* | *Hs99999904_m1* | *HKG-3* |
