# Supplementary material for: Transcriptomic population markers for human population discrimination
Source: BMC Genet. 2018 Aug 7;19:54. doi: 10.1186/s12863-018-0663-2 (PMC6081795; doi:10.1186/s12863-018-0663-2)
Supplement: Supplementary file 11 — : Table S5. Primer design for qRT-PCR validation experiment. (DOCX 13 kb) [file 12863_2018_663_MOESM11_ESM.docx]

**Additional file 11: Table S5.** Primer design for qRT-PCR validation experiment.

| **Gene name** | **Genbank Number** | **Primer F**  **sequence** | **Primer R**  **sequence** | **Probe sequence** | **Amplicon length (bp)** |
| --- | --- | --- | --- | --- | --- |
| *UTS2* | NM_006786.3 | ctcctgctgtttgcttttcat | gcgtcttcatgaggtgctg | 55 tcctctcc | 105 |
| *UGT2B17* | NM_001077.2 | tgtcagaagaaagtgccaaca | ggttctatggagatttgatggc | 61 ttgcccag | 77 |
